# Patient‐reported outcome and survival in premenopausal hormone receptor‐positive breast cancer patients at moderate to high risk: comparing toremifene with aromatase inhibitor in a real‐world study

**DOI:** 10.1002/mco2.698

**Published:** 2024-09-15

**Authors:** Yaping Yang, Fengxia Gan, Ting Luo, Qun Lin, Wenqian Yang, Lili Chen, Wei Zhang, Qiang Liu, Chang Gong

**Affiliations:** ^1^ Guangdong Provincial Key Laboratory of Malignant Tumor Epigenetics and Gene Regulation, Guangdong‐Hong Kong Joint Laboratory for RNA Medicine Sun Yat‐sen Memorial Hospital, Sun Yat‐sen University Guangzhou Guangdong China; ^2^ Breast Tumor Center Sun Yat‐Sen Memorial Hospital, Sun Yat‐Sen University Guangzhou Guangdong China; ^3^ Institute for Breast Health Medicine, Cancer Center, Breast Center West China Hospital, Sichuan University Chengdu Sichuan China

**Keywords:** aromatase inhibitor, patient‐reported outcome, premenopausal hormone receptor‐positive breast cancer, toremifene

## Abstract

Toremifene, a selective estrogen receptor modulator, is commonly used in China for premenopausal breast cancer patients. This real‐world study aimed to compare patient‐reported outcome (PRO) and survival between toremifene and aromatase inhibitor (AI) plus ovarian function suppression (OFS) in patients with moderate‐/high‐risk premenopausal hormone receptor (HR)‐positive breast cancer. The primary endpoint was PROs, assessed using SF‐36 and EQ‐5D‐5L questionnaires between January and March 2023. A total of 392 patients were included, with 171 receiving toremifene and 221 receiving AI. The toremifene group showed significantly higher scores in the role physical (*p* = 0.034) and mental health (*p *= 0.009) dimensions of SF‐36 and lower anxiety/depression (AD) scores (*p *= 0.038) in EQ‐5D‐5L compared to AI group. The estimated 5‐ and 8‐year disease‐free survival (DFS) rates were similar in toremifene and AI groups: 96.5% versus 91.9%, and 87.4% versus 87.8% (*p *= 0.39), respectively. Adverse event rates were similar in two groups, except for a greater risk of endometrial thickening (*p *< 0.001) and a lower occurrence of morning stiffness (*p *< 0.001) in the toremifene compared to the AI group. Premenopausal HR‐positive breast cancer patients receiving toremifene plus OFS had better role physical and mental health outcomes and lower AD dimensions than those receiving AI plus OFS. Both treatments had comparable DFS and favorable tolerability profiles.

## Graphical abstract

This real‐world study aimed to compare patient‐reported outcome (PRO) and survival between toremifene and aromatase inhibitor (AI) plus ovarian function suppression (OFS) in patients with moderate‐/high‐risk premenopausal hormone receptor (HR)‐positive breast cancer. A total of 392 patients were included, with 171 receiving toremifene and 221 receiving AI. Our study showed that patients receiving toremifene plus OFS demonstrated significantly elevated scores in the role physical and general health dimensions on SF‐36, along with a less severe AD dimension on EQ‐5D‐5L, compared to those undergoing AI plus OFS. Both treatments had comparable disease‐free survival (DFS) and favorable tolerability profiles.

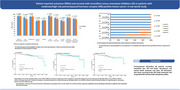

## 1 INTRODUCTION

According to GLOBOCAN 2022, 357,161 new cases and 74,986 deaths of female breast cancer were estimated to occur in 2022, ranking second for incidence and sixth for mortality.^1^ The average age of breast cancer diagnosis of in China is 48–50 years, which is more than 10 years younger than reported ages in Western countries, with a predominance of premenopausal cases constituting the majority of breast cancer patients.^2^, ^3^ Hormone receptor (HR)‐positive breast cancer, which accounts for about two‐thirds of cases, emphasizes the significance of endocrine therapy in its treatment. Selective estrogen receptor modulators (SERMs) such as tamoxifen and toremifene demonstrate comparable efficacy in treating premenopausal HR‐positive breast cancer.^4^, ^5^, ^6^

Studies have revealed that CYP2D6 *10 gene mutation affects tamoxifen treatment effectiveness for breast cancer. Notably, individuals with CYP2D6 *10 T/T genotype experience less favorable clinical outcomes when undergoing adjuvant tamoxifen.^7^, ^8^ The CYP2D6 *10 polymorphism is commonly observed in Chinese population. An analysis of patient data from China e National Cancer Center revealed that adjuvant endocrine therapy with toremifene is more effective for CYP2D6 *10 T/T breast cancer patients.^9^ Consequently, toremifene has gained widespread clinical usage in China.

Several large‐scale long‐term follow‐up studies, including ABCSG XII, ASTRRA, TEXT, SOFT, and ZIPP underscore the benefits of ovarian function suppression (OFS) when combined with an aromatase inhibitor (AI) or tamoxifen for premenopausal HR‐positive breast cancer patients.^10^, ^11^, ^12^, ^13^ The 2022 ESMO BCY5 guidelines recommend the combination of OFS and AI as the primary choice for patients with a high risk of recurrence, while OFS plus tamoxifen should be the preferred choice for patients who are susceptible to AI toxicity.^14^ Similarly, the 2019 Asian Breast Cancer Cooperative Group consensus for Asian populations also recommends the use of OFS plus tamoxifen or OFS plus AI for the treatment of postmenopausal early HR‐positive patients at moderate to high risk.^15^

As the efficacy of breast cancer treatment continues to improve, survival rates have increased, and the focus on patient health‐related quality of life (HRQoL) has grown in clinical research and treatment.^15^ Patients undergoing endocrine therapy may experience adverse reactions such as vaginal symptoms, endometrial cancer, venous thromboembolism, cerebrovascular events, musculoskeletal symptoms, and sexual dysfunction, all of which can detrimentally impact their quality of life (QoL).^16^, ^17^, ^18^, ^19^ As well as clinically reported adverse events (AEs), HRQoL questionnaires are also used as important tools to measure breast cancer patients’ HRQoL.^20^ Several HRQoL scales are commonly utilized used in breast cancer research, including EORTC QLQ C30, EORTC BR‐23, FACT‐B, WHO‐QOL‐BREF, IBCSG, MENQOL, FACT/FACIT, SF‐36, and EQ‐5D‐5L.^21^, ^22^, ^23^, ^24^ Previous studies have yield varied results regarding the QoL among early breast cancer patients when comparing tamoxifen to AIs. N‐SAS BC 03^24^ and N‐SAS BC 04^26^ HRQOL studies in Japanese postmenopausal patients indicate that HRQOL, as measured by FACT‐B, is better in patients treated with tamoxifen than in those receiving exemestane or anastrozole. Conversely, the NSABP B‐35^27^ study conducted in Canada, Mexico, and the United States found no significant difference in SF‐12 physical health component and mental health (MH) between anastrozole and tamoxifen treatment groups. Park et al.^28^ utilized SEER‐MHOS data file and US Medicare population‐based data to investigate HRQoL disparities among various adjuvant endocrine therapies, including tamoxifen, anastrozole, letrozole, and exemestane. The findings revealed that there were no significant advantages or disadvantages associated with the four different types of adjuvant endocrine therapies in terms of physical component summary. However, patients receiving tamoxifen and anastrozole demonstrated significantly superior mental component summary scores compared to those receiving letrozole and exemestane.

To date, there exists no study specifically addressing the impact of toremifene on QoL of Chinese premenopausal breast cancer patients and comparing it with AI. We aimed to assess the impact of toremifene treatment in comparison to AI on QoL, efficacy, and safety by collecting patient‐reported outcome (PRO) data in a real‐world setting. Our study found that patients who received toremifene plus OFS had better PRO than those receiving AI plus OFS. There were no significant differences in disease‐free survival (DFS) between the toremifene and AI groups. Given the widespread use of both toremifene and AIs in clinical practice, our results have important implications for personalized treatment in these patients.

## 2 RESULTS

### 2.1 Patients’ characteristics

A total of 392 patients were included, with 171 (43.6%) receiving toremifene and 221 (56.4%) receiving AI. At diagnosis, the median age was 39 (range, 21–54) years, and most patients aged <40 years. The median follow‐up reached 68 months. The majority of patients were at T1 stage (56.1%), stage II (46.4%) had histological grade I–II (49.7%), and presented with no lymph node involvement (44.4%). Among the patients, 309 (78.8%) were HER2‐negative, 359 (91.6%) had a baseline estrogen receptor (ER) expression of over 20%, 201 (51.3%) were at moderate risk, and 348 (88.8%) received chemotherapy. Compared with AI group, toremifene group exhibited a younger age, a higher proportion of patients without lymph node metastasis, at stage I, HER2‐negative, with ER expression of ≤20%, at moderate risk, and without chemotherapy (Table 1).

**TABLE 1:** Baseline characteristics of included patients in toremifene and AI groups.

	Total (*n* = 392) *N* (%)	Toremifene (*n* = 171) *N* (%)	AI (*n* = 221) *N* (%)	*p* value
First diagnosed age				0.009^**^
<40	200 (51.0)	100 (58.5)	100 (45.2)	
≥40	192 (49.0)	71 (41.5)	121 (54.8)	
HER2				0.041^*^
Negative	309 (78.8)	143 (83.6)	166 (75.1)	
Positive	83 (21.2)	28 (16.4)	55 (24.9)	
ER				<0.001^***^
≤20%	33 (8.4)	24 (14.0)	9 (4.1)	
>20%	359 (91.6)	147 (86.0)	212 (95.9)	
T stage				0.095
T1	220 (56.1)	105 (61.4)	115 (52.0)	
T2	150 (38.3)	60 (35.1)	90 (40.7)	
T3–4	22 (5.6)	6 (3.5)	16 (7.2)	
N stage				<0.001^***^
N0	174 (44.4)	109 (63.7)	65 (29.4)	
N1	136 (34.7)	45 (26.3)	91 (41.2)	
N2–3	82 (20.9)	17 (9.9)	65 (29.4)	
Stage				<0.001^***^
I	127 (32.4)	79 (46.2)	48 (21.7)	
II	182 (46.4)	72 (42.1)	110 (49.8)	
III	83 (21.2)	20 (11.7)	63 (28.5)	
Histological grade				0.707
I–II	195 (49.7)	85 (49.7)	110 (49.8)	
III	121 (30.9)	50 (29.2)	71 (32.1)	
Unknown	76 (19.4)	36 (21.1)	40 (18.1)	
Risk classification				<0.001^***^
Moderate	201 (51.3)	120 (70.2)	81 (36.7)	
High	191 (48.7)	51 (29.8)	140 (63.3)	
Chemotherapy				<0.001^***^
Yes	348 (88.8)	138 (80.7)	210 (95.0)	
No	43 (11.0)	32 (18.7)	11 (5.0)	
Unknown	1 (0.3)	1 (0.6)	0 (0.0)	

Abbreviations: AI, aromatase inhibitor; ER, estrogen receptor; HER2, human epidermal growth factor receptor 2.

^*^ *p* < 0.05; ^**^ *p* < 0.01; ^***^ *p* < 0.001.

### 2.2 PROs

The toremifene group exhibited higher scores in the role physical (RP) and MH dimensions in SF‐36 compared to the AI group (85.96 ± 30.2 vs. 78.73 ± 36.93, *p* = 0.034 and 71.6 ± 15.78 vs. 67.11 ± 17.33, *p* = 0.009, respectively). However, no significant differences were found in the other dimensions of SF‐36 between the two groups (*p* > 0.05; Table 2).

**TABLE 2:** Raw scores and standard scores of SF‐36 between toremifene and AI groups.

Dimensions	Raw scores		Standard scores		*p* value
	Toremifene	AI	Toremifene	AI	
Physical functioning	28.40 ± 1.97	28.35 ± 2.60	91.99 ± 9.83	91.76 ± 12.98	0.851
Role physical	7.44 ± 1.21	7.15 ± 1.48	85.96 ± 30.20	78.73 ± 36.93	0.034^*^
Bodily pain	10.97 ± 1.19	11.02 ± 1.32	89.70 ± 11.95	90.21 ± 13.19	0.693
General health	18.75 ± 2.35	19.25 ± 2.85	68.77 ± 11.76	71.27 ± 14.24	0.058
Validity	18.15 ± 3.16	18.26 ± 3.60	70.76 ± 15.82	71.31 ± 18.01	0.751
Social function	9.37 ± 1.18	9.21 ± 1.25	92.18 ± 14.79	90.10 ± 15.64	0.183
Role emotional	5.50 ± 0.97	5.38 ± 1.05	83.24 ± 32.21	79.34 ± 35.11	0.259
Mental health	22.90 ± 3.95	21.78 ± 4.33	71.60 ± 15.78	67.11 ± 17.33	0.009^**^
Physical component summary	84.11 ± 12.10	82.99 ± 13.66	84.11 ± 12.10	82.99 ± 13.66	0.394
Mental component summary	79.44 ± 15.36	76.97 ± 16.57	79.44 ± 15.36	76.97 ± 16.57	0.130

Abbreviation: AI, aromatase inhibitor.

^*^ *p* < 0.05; ^**^ *p* < 0.01.

**FIGURE 1:**
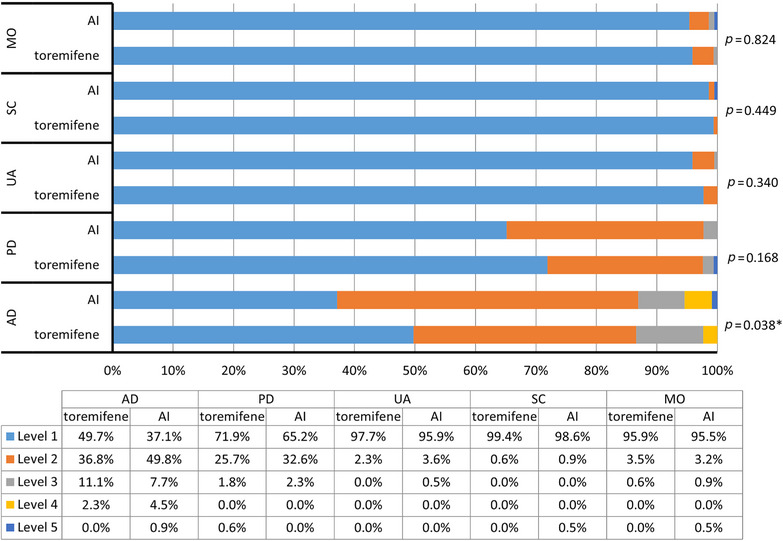
EQ‐5D‐5L frequencies and proportions reported by dimension and level between toremifene and AI groups. Level 1, no problems; Level 2, slight problems; Level 3, moderate problems; Level 4, severe problems; Level 5, extreme problems/ unable to do. AD, anxiety/depression; AI, aromatase inhibitor; MO, mobility; PD, pain/discomfort; SC, self‐care; UA, usual activities. ^*^ *p* < 0.05.

Significant differences were observed in the distribution of responses to the AD dimension of EQ‐5D‐5L between the toremifene and AI groups (mean rank: 184.14 vs. 206.07, *p* = 0.038), with the proportions of patients reported “no problems” were 49.7% and 37.1%, respectively. No significant differences were observed in the other dimensions of EQ‐5D‐5L between the two groups, with the majority patients reported “no problems” (95.9% vs. 95.5% in mobility, 99.4% vs. 98.6% in self‐care, 97.7% vs. 95.9% in usual activities, 71.9% vs. 65.2% in pain/discomfort in the toremifene and AI groups, respectively; Figure 1). There was no significant difference in EQ‐5D‐5L index (0.94 ± 0.07 vs. 0.92 ± 0.09, *p* = 0.053) between the two groups.

### 2.3 DFS

**FIGURE 2:**
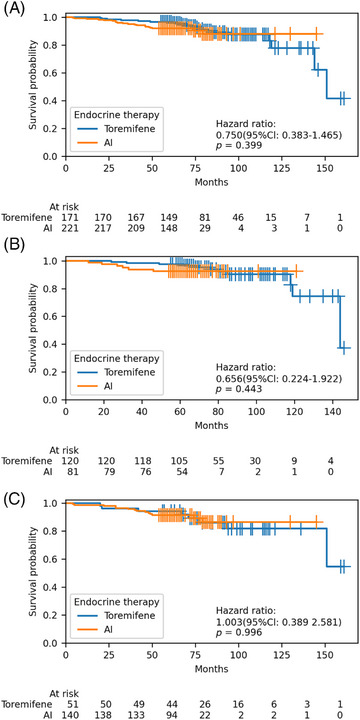
Kaplan–Meier curve of disease‐free survival (DFS). (A) Kaplan–Meier curve of DFS for different endocrine therapy treatments in all patients. Comparison between aromatase inhibitor (AI) and toremifene groups. (B) Kaplan–Meier curve of DFS for different endocrine therapy treatments in moderate‐risk patients. Comparison between AI and toremifene groups. (C) Kaplan–Meier curve of DFS for different endocrine therapy treatments in high‐risk patients. Comparison between AI and toremifene groups.

**FIGURE 3:**
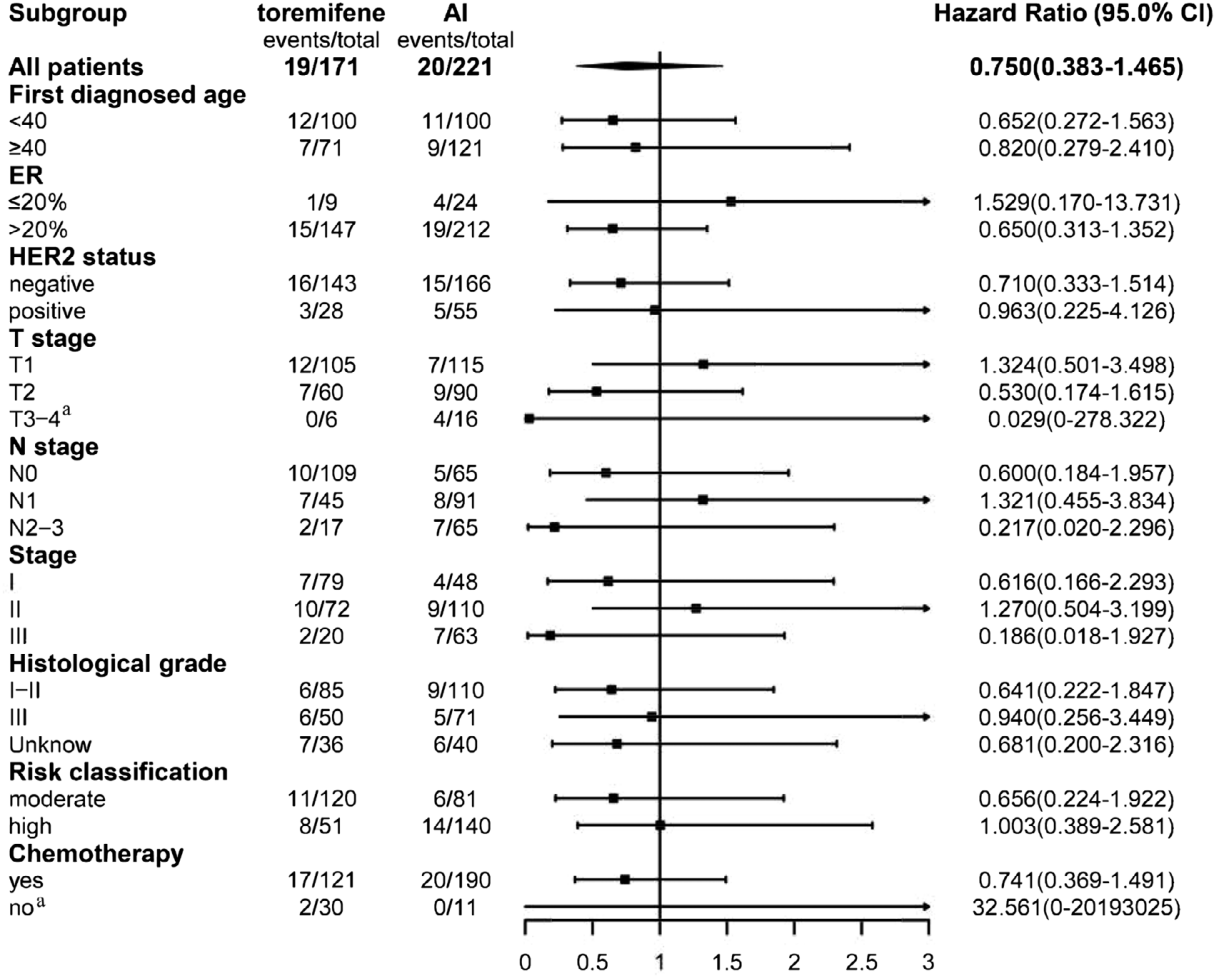
Hazard ratio of subgroup univariate cox analysis in disease‐free survival (DFS) in all patients and subgroups. AI, aromatase inhibitor; ER, estrogen receptor; HER2, human epidermal growth factor receptor 2. ^a^The coefficients of this cox regression did not converge.

**FIGURE 4:**
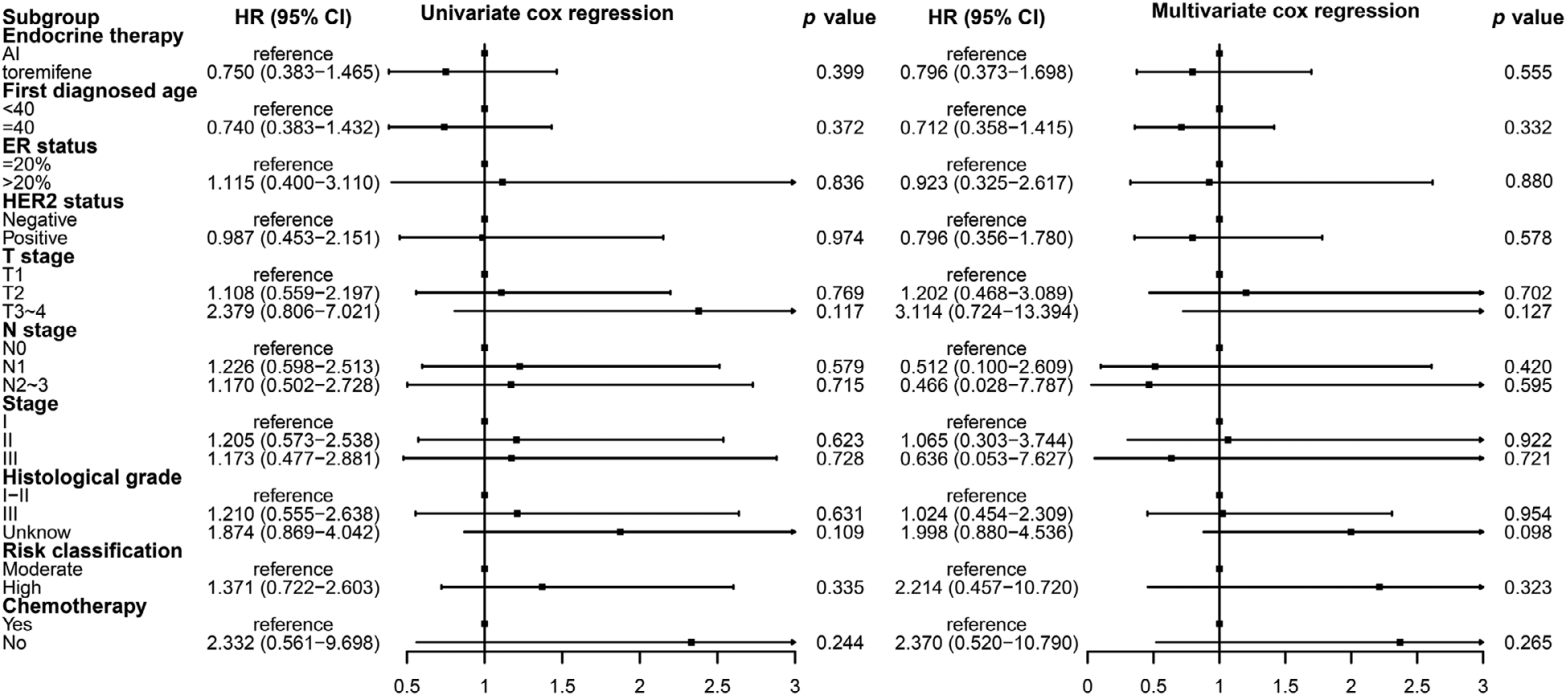
Hazard ratio of univariate cox regression and multivariate cox regression in disease‐free survival (DFS). AI, aromatase inhibitor; ER, estrogen receptor; HER2, human epidermal growth factor receptor 2.

There were 39 breast cancer recurrences or metastasis events. The estimated 5‐ and 8‐year DFS rates were similar between the toremifene and AI groups: 96.5% (95% confidence interval [CI]: 93.7–99.2) and 91.9% (95% CI: 88.2–95.5) at 5 years, and 87.4% (95% CI: 81.1–93.8) and 87.8% (95% CI: 81.1–94.4) at 8 years, respectively. Between the two groups, a hazard ratio for DFS was 0.75 (95% CI: 0.38–1.46), *p* = 0.39. Stratification by various factors, including age (<40 years vs. ≥40 years), HER2 status (negative vs. positive), ER expression level (≤20% vs. >20%), T stage (T1 vs. T2 vs. T3–4), N stage (N0 vs. N1 vs. N2–3), stage (I vs. II vs. III), histological grade (I–II vs. III vs. unknown), risk classification (moderate vs. high), and chemotherapy (yes vs. no), DFS remained comparable between the two groups (Table 3 and Figures 2 and 3). Both univariate and multivariate Cox regression analyses did not reveal any significant associations between these factors and DFS (Figure 4).

**TABLE 3:** DFS at 5 years and 8 years in toremifene and AI groups for different prognostic factors.

	DFS at 5 years		DFS at 8 years	
	Toremifene	AI	Toremifene	AI
All patients	96.5 (93.7–99.2)	91.9 (88.2–95.5)	87.4 (81.1–93.8)	87.8 (81.1–94.4)
First diagnosed age				
<40	96.0 (92.2–99.8)	90.0 (84.1–95.9)	86.4 (78.1–94.6)	87.1 (79.1–95.1)
≥40	97.2 (93.3–100.0)	93.4 (89.0–97.8)	89.8 (80.8–98.9)	88.1 (77.1–99.0)
HER2 status				
Negative	96.5 (93.4–99.5)	92.1 (88.0–96.2)	87.9 (81.0–94.8)	86.6 (78.1–95.1)
Positive	96.4 (89.6–103.3)	90.9 (83.3–98.5)	84.2 (67.0–100.0)	90.9 (83.3–98.5)
ER status				
≤20%	87.5 (74.3–100.0)	88.9 (68.4–109.4)	87.5 (74.3–100.7)	88.9 (68.4–100.0)
>20%	98.0 (95.7–100.0)	92.0 (88.3–95.6)	86.6 (79.0–94.2)	87.5 (80.3–94.6)
T stage				
T1	94.3 (89.8–98.7)	93.9 (89.5–98.3)	87.8 (80.2–95.3)	93.9 (89.5–98.3)
T2	100.0 (100.0–100.0)	91.1 (85.2–97)	86.5 (74.9–98.0)	87.1 (77.7–96.6)
T3–4	100.0 (100.0–100.0)	81.3 (62.1–100.0)	100.0 (100.0–100.0)	NA
N stage				
N0	97.2 (94.2–100.0)	92.3 (85.8–98.8)	88.9 (81.1–96.7)	92.3 (85.8–98.8)
N1	93.3 (86–100.0)	92.3 (86.8–97.8)	84.2 (72.3–96.1)	89.5 (81.9–97.1)
N2–3	100.0 (100.0–100.0)	90.8 (83.7–97.8)	90 (71.4–100.0)	83.8 (69.1–98.5)
Stage				
I	96.2 (92.0–100.0)	91.7 (83.8–99.5)	90.6 (82–99.2)	91.7 (83.8–99.5)
II	95.8 (91.2–100.0)	92.7 (87.9–97.6)	84.4 (74.8–94.1)	90.2 (83.4–97.0)
III	100.0 (100.0–100.0)	90.5 (83.2–97.7)	90.9 (73.9–100.0)	82.9 (67.3–98.6)
Histological grade				
I–II	97.6 (94.4–100.0)	92.7 (87.9–97.6)	91.9 (84.7–99.0)	89.6 (81.9–97.2)
III	98.0 (94.1–100.0)	93.0 (87.0–98.9)	85.2 (72.6–97.9)	93.0 (87.0–98.9)
Unknown	91.7 (82.6–100.0)	87.5 (77.3–97.7)	81.3 (65.4–97.1)	76.6 (54.6–98.5)
Risk classification				
Moderate	97.5 (94.7–100.0)	92.6 (86.9–98.3)	90.2 (83.3–97.1)	92.6 (86.9–98.3)
High	94.1 (87.7–100.0)	91.4 (86.8–96.1)	81.9 (69.0–94.7)	86.1 (77.5–94.6)
Chemotherapy				
Yes	96.4 (93.3–99.5)	91.4 (87.6–95.2)	86.5 (79.4–93.7)	87.0 (80.0–94.1)
No	96.9 (90.8–100.0)	100.0 (100.0–100.0)	92.8 (83.0–102.5)	100.0 (100.0–100.0)

Abbreviations: AI, aromatase inhibitor; DFS, disease‐free survival; ER, estrogen receptor; HER2, human epidermal growth factor receptor 2.

### 2.4 Safety

A total of 310 (79.1%) patients reported AEs, with 129 patients (75.4%) in the toremifene group and 181 patients (81.9%) in the AI group. However, AEs in the two groups did no differ statistically significant (*p* = 0.133). The most common AEs both in two group were arthralgia [64 (37.4%) in the toremifene group vs. 84 (38.0%) in the AI group], facial flushing [40 (23.4%) in the toremifene group vs. 45 (20.4%) in the AI group], vaginal dryness [40 (23.4%) in the toremifene group vs. 60 (27.1%) in the AI group], myalgia [33 (19.3%) in the toremifene group vs. 35 (15.8%) in the AI group]. Among the reported AEs, endometrial thickening showed a higher incidence in the toremifene group compared to AI group (17.5% vs. 1.8%, *p* < 0.001), while morning stiffness had a lower incidence (7.6% vs. 14.5%, *p* = 0.034; Table 4).

**TABLE 4:** Adverse events.

	Toremifene (*n* = 171)	AI (*n* = 221)	*p* value
Amenorrhea or menstrual disorder	12 (7)	15 (6.8)	0.929
Gastrointestinal discomfort	5 (2.9)	3 (1.4)	0.304
Tidal fever	13 (7.6)	17 (7.7)	0.973
Increased secretion	1 (0.6)	1 (0.5)	1
Recurrent urinary infections	0 (0)	1 (0.5)	1
Osteoporosis, pain	21 (12.3)	34 (15.4)	0.380
Dyslipidemia	6 (3.5)	13 (5.9)	0.278
Endometrial thickening	30 (17.5)	4 (1.8)	<0.001^***^
Ovarian cyst	1 (0.6)	0 (0)	0.436
Myoma of uterus	3 (0.9)	2 (1.8)	0.657
Leukopenia	0 (0)	1 (0.5)	1
Elevated transaminase	1(0.6)	5 (2.3)	0.238
Hysterectomy and adnexectomy	4 (2.3)	1 (0.5)	0.172
Facial flushing	40 (23.4)	45 (20.4)	0.470
Cold sweat	24 (14)	19 (8.6)	0.088
Night sweat	21 (12.3)	26 (11.8)	0.876
Vaginal itching	19 (11.1)	17 (7.7)	0.245
Abnormal vaginal discharge	8 (4.7)	4 (1.8)	0.102
Vaginal bleeding	5 (2.9)	3 (1.4)	0.304
Vaginal dryness	40 (23.4)	60 (27.1)	0.397
Arthralgia	64 (37.4)	84 (38.0)	0.906
Morning stiffness	13 (7.6)	32 (14.5)	0.034^*^
Myalgia	33 (19.3)	35 (15.8)	0.369
Carpal tunnel syndrome	8 (4.7)	4 (1.8)	0.102
Trigger finger	3 (1.8)	8 (3.6)	0.361
Decreased grip strength	6 (3.5)	17 (7.7)	0.080

Abbreviation: AI, aromatase inhibitor.

^*^ *p* < 0.05; ^***^ *p* < 0.001.

In this study, AE was based on patient self‐report, and no patients reported serious adverse event (SAE). No patients discontinued endocrine drug due to AEs. When they had some AEs due to the endocrine treatment, they changed the treatment drug from toremifene to AI (7.02%, 12/171), or from AI to toremifene (18.10%, 40/221).

## 3 DISCUSSION

To our knowledge, this study is the first in China to explore PROs and survival outcomes in premenopausal HR‐positive breast cancer patients at moderate to high risk who were treated with toremifene or AI combined with OFS. Our findings reveal that patients receiving toremifene plus OFS demonstrated significantly elevated scores in the RP and general health (GH) dimensions on SF‐36, along with a less severe AD dimension on EQ‐5D‐5L, compared to those undergoing AI plus OFS. DFS did not significantly differ between toremifene and AI groups. Regarding safety, the toremifene group had a higher occurrence of endometrial thickening than the AI group, while the occurrence of morning stiffness was lower. The potential implication of this finding was that in this 5 years long‐term endocrine therapy, toremifene combined with OFS was a viable option for the treatment with better PROs and similar survival outcomes compared with AI combined with OFS in Chinese patients with moderate‐/high‐risk premenopausal HR‐positive breast cancer.

In this study, we found the two treatment groups had similar 5‐ and 8‐year DFS rates, without any significant differences. Stratified analyses based on various factors such as age, HER2 status, ER, postoperative stage, lymph node metastasis, and risk classification also showed no difference in DFS between the two groups. Previous studies have not directly compared toremifene with AI in premenopausal patients. The NAFTA study^6^ reported similar 5‐year DFS rates between tamoxifen and toremifene in perimenopausal and postmenopausal breast cancer patients (91.2% vs. 91.2%). Retrospective studies have shown that toremifene has similar efficacy to tamoxifen in premenopausal patients.^5^ However, a study found that toremifene was superior to tamoxifen in adjuvant endocrine treatment for CYP2D6 *10 T/T breast cancer patients.^8^ In contrast to the findings of TEXT and SOFT studies, which showed that in premenopausal patients, the combination of exemestane with OFS significantly improved both 5‐ and 8‐year DFS compared to the combination of tamoxifen with OFS [5‐year DFS rates: exemestane vs. tamoxifen were 91.1% vs. 87.3%, with a HR of 0.72 (95% CI: 0.60–0.85); 8‐year DFS rates: 86.8% vs. 82.8%, with a HR of 0.77 (95% CI: 0.67–0.90)],^29^, ^30^ our study did not found such distinctions. This discrepancy could be attributed to this study's specific focus on Chinese premenopausal women with breast cancer, as opposed to the diverse global population included in TEXT and SOFT studies, introducing potential racial differences. Chemotherapy was used as a stratification factor in TEXT and SOFT studies and was administered to 57.4%, as compared with 88.8% in our study. In addition, the patients in our study were younger (proportion of <40 years old 51.0% vs. 26.9%) and had a higher proportion of HER2‐positive patients (21.2% vs. 12.1%), lower proportion of negative lymph nodes patients (44.4% vs. 57.8%), lower proportion of T1 stage (56.1% vs. 62.3%), lower proportion of histological grade I–II (49.7% vs. 74.5%) than those in the TEXT and SOFT studies.

We found that toremifene was superior to AI in the domain of the role physical of SF‐36 scale, without any difference in physical component summary. Our findings contrast with a prior study that demonstrated tamoxifen's superiority over AI results in overall HRQoL scores in Japanese breast cancer patients.^25^, ^26^ One possible explanation for this inconsistency is that our study focused on premenopausal patients, whereas previous studies included perimenopausal or postmenopausal patients. We also found that toremifene was superior to AI in the domain of MH in SF‐36 and had a lower severity of AD in the EQ‐5D‐5L. These results indicate that toremifene is possibly superior to AI in terms of QoL in MH. Our findings are consistent with a previous study utilizing SEER‐MHOS data and US Medicare population‐based data, which reported tamoxifen's superiority over letrozole and exemestane in terms of mental component of HRQoL.^28^ But in China, more patients chose toremifene than tamoxifen because of the CYP2D6*10 gene mutation, which has racial and geographical differences, with a higher prevalence in Asian patients compared to Western populations. The CYP2D6*10 genotype is associated with lower levels of active metabolites of tamoxifen in Asian breast cancer patients, which may impact the clinical effectiveness of tamoxifen^31^, ^32^, ^33^ and had poor prognosis.^9^ In the TEXT and SOFT studies, there is no clear evidence supporting the superiority of exemestane plus OFS or tamoxifen plus OFS from the QoL perspective. Currently, there are no studies on the QoL with the combination of toremifene and OFS. Compared to patients receiving tamoxifen plus OFS, patients receiving exemestane plus OFS had a more noticeable increase in bone or joint pain, especially in the short term.^34^ Currently, there are no studies on the QoL with the combination of toremifene and OFS.

In terms of safety, the main AEs reported with toremifene plus OFS included arthralgia, vaginal symptoms, facial flushing, myalgia, and endometrial thickening. Similarly, arthralgia, vaginal symptoms, facial flushing, myalgia, and osteoporosis were the main AEs observed with AI plus OFS. The toremifene exhibited a higher occurrence of endometrial thickening than AI, while the occurrence of morning stiffness was lower. In a previous study from China, the incidence of endometrial thickening with single‐agent toremifene was lower than that with tamoxifen. In 212 premenopausal patients receiving adjuvant treatment with toremifene, 22 cases of endometrial abnormalities were observed.^35^ These findings align with prior studies.^18^, ^19^, ^36^ However, multiple studies have shown that the occurrence of endometrial cancer is negligible or absent.^5^, ^37^ Current research discusses the possibility that endometrial thickening in users of antiestrogens may not necessarily be a sign of endometrial hyperplasia.^38^ Endometrial thickening may also be the result of enlargement of subendometrial glands.^39^ Currently, in Chinese diagnosis and treatment guidelines or consensus, monitoring and management of endometrial thickening are recommended for both premenopausal and postmenopausal women^40^ which may impact on patient QoL.

This study has some limitations. First, in this real‐world study, the sample size and the number of events observed are relatively small, which might limit the statistical power to detect significant differences or might affect the generalizability of the findings. Second, it is the cross‐sectional design impedes the prospective collection of PRO at different treatment time points. Previous studies have found that PRO may change over the course of treatment,^41^ necessitating the need for a multicenter and prospective study. Additionally, a study found that tamoxifen exhibited superior HRQoL compared to letrozole and exemestane, but not anastrozole, suggesting that the choice of AI may impact HRQoL.^28^ Future studies conducted specifically among the Chinese population could provide valuable insights into distinguishing between the different AIs. The monarchE study has established the superior efficacy of adding abemaciclib to endocrine therapy in high‐risk HR‐positive breast cancer patients.^42^ Abemaciclib has obtained approval from National Medical Products Administration (NMPA) and Food and Drug Administration (FDA) for use in high‐risk HR‐positive breast cancer patients. This implies that the optimal treatment for some individuals included in our study may have changed. Therefore, future studies should investigate PRO outcomes in patients receiving cyclin‐dependent kinase 4 and 6 inhibitors plus endocrine therapy.

## 4 CONCLUSION

In conclusion, this real‐world study represents the first analysis of PROs and survival outcomes associated with toremifene or AI combined with OFS in Chinese patients with moderate‐/high‐risk premenopausal HR‐positive breast cancer. Our findings indicate that patients receiving toremifene plus OFS had better RP and MH dimensions on SF‐36, as well as less severe AD dimensions on EQ‐5D‐5L compared to those receiving AI plus OFS. No significant differences in 5‐ or 8‐year DFS were observed between the toremifene and AI groups. Both treatments were generally well tolerated. Toremifene emerges as a viable option for the treatment of Chinese patients with moderate‐/high‐risk premenopausal HR‐positive early breast cancer.

## 5 METHODS

### 5.1 Study design and patients

This is a real‐world study, as a cross‐sectional study. Premenopausal patients with HR‐positive, moderate‐/high‐risk (non–low‐risk) breast cancer who received OFS combined with either toremifene or AI between January 1, 2010 and December 31, 2017 were enrolled at the Breast Cancer Center of Sun Yat‐sen Memorial Hospital. The clinical characteristics data of patients were obtained from the Breast Cancer Center database of Sun Yat‐sen Memorial Hospital, Sun Yat‐sen University. The PROs and AEs information were collected through questionnaires. The survival information was derived from a comprehensive analysis of both the database and questionnaires.

Patients must fulfill all the following criteria to be eligible for this study. (1) women whose age ≥18 years old and signed informed consent; (2) breast cancer patients with premenopausal status at the time of curative breast cancer surgery; (3) patients underwent at least 5 years endocrine therapy; (4) patients with HR‐positive moderate‐ or high‐risk breast cancer; (5) patients with early‐stage breast cancer at the time of radical breast cancer surgery and at the time of curative breast cancer surgery; low‐risk breast cancer was defined by meeting all of the following specific criteria, including tumor size (pT) ≤2 cm, histological grade I, absence of vascular tumor thrombus, positive for ER and/or progesterone receptor (PR), HER2‐negative, and age of 35 years or older. So, HR‐positive moderate‐ or high‐risk breast cancer was defined as non–HR‐positive low‐risk breast cancer. HER2‐negative is defined as immunohistochemistry (IHC) 0 or 1+. If IHC is 2+, a negative fluorescence in situ hybridization (FISH) test is required to confirm the HER2‐negative status. Baseline information, treatment information, and breast cancer‐related information were obtained from the existing breast cancer registry. Questionnaires were administered to collect information on adverse drug reactions and QoL using SF‐36 and EQ‐5D‐5L from January 1 to March 31, 2023.

### 5.2 Endpoints

The primary endpoint was PROs, which were assessed using SF‐36 and EQ‐5D‐5L. Disease‐free survival (DFS) and safety were secondary endpoints. DFS, defined as the time from the surgery until any relapse, secondary malignancy, or death from any cause.

SF‐36 is widely validated scales used globally to evaluate QoL in various languages. A diverse assessment tool with 36 questions divided into eight domains: physical functioning (PF, 10 questions), GH (five questions), RP (four questions, representing role restrictions because of physical health issues), bodily pain (BP, two questions), social functioning (SF, two questions), vitality (VT, four questions), role emotional (RE, three questions, indicating role constraints because of emotional issues), and MH (five questions). Each domain is scored on a scale (0–100), where higher scores signify better health. Physical component summary is derived from the domains of PF, GH, RP, and BP, while mental component summary is derived from SF, VT, RE, and MH.^43^, ^44^, ^45^

EQ‐5D‐5L consists of five dimensions: mobility, self‐care, usual activities, pain and discomfort, and anxiety/depression (AD). There are five levels of response in individual dimension: “1” signifies “no problems,” “2” represents “slight problems,” “3” corresponds to “moderate problems,” “4” denotes “severe problems,” and “5” is indicative of “unable to/extreme problems.”. EQ‐5D‐5L states can be converted to EQ‐5D‐5L index values to summary health state by standard EQ‐5D‐5L value sets.^46^, ^47^

### 5.3 Statistical analysis

The comparisons of baseline characteristics between these two groups were calculated using chi‐square test. The correlation between treatment group and SF‐36 scores was assessed using Student's *t*‐test, while the association between treatment group and EQ‐5D‐5L scores was analyzed using nonparametric tests. DFS was analyzed by Kaplan–Meier and Cox regression. Safety was presented with number and percentage and analyzed using chi‐square test between treatment groups. SPSS software (version 22.0) was used for statistical analysis. Statistical tests were performed as two‐sided and judged significant at *p* < 0.05.

## AUTHOR CONTRIBUTIONS

Chang Gong and Qiang Liu contributed to study concept and design. Yaping Yang contributed to study concept, design, and writing. Fengxia Gan contributed to study analysis and writing. Ting Luo contributed to writing. Qun Lin, Wenqian Yang, Lili Chen, and Wei Zhang contributed to data collections. All the authors read and approve the final manuscript.

## CONFLICT OF INTEREST STATEMENT

The authors declare no conflicts of interest.

## ETHICS STATEMENT

The study was approved by the Medical Ethics Committee, Sun Yat‐sen Memorial Hospital, Sun Yat‐sen University ((approval number: SYSKY‐2022‐445‐02). Written informed consent was obtained from all participants.

## Data Availability

Data generated or analyzed during the study are available from the corresponding author by request.
